# iT3SE-PX: Identification of Bacterial Type III Secreted Effectors Using PSSM Profiles and XGBoost Feature Selection

**DOI:** 10.1155/2021/6690299

**Published:** 2021-01-06

**Authors:** Chenchen Ding, Haitao Han, Qianyue Li, Xiaoxia Yang, Taigang Liu

**Affiliations:** College of Information, Shanghai Ocean University, Shanghai 201306, China

## Abstract

Identification of bacterial type III secreted effectors (T3SEs) has become a popular research topic in the field of bioinformatics due to its crucial role in understanding host-pathogen interaction and developing better therapeutic targets against the pathogens. However, the recognition of all effector proteins by using traditional experimental approaches is often time-consuming and laborious. Therefore, development of computational methods to accurately predict putative novel effectors is important in reducing the number of biological experiments for validation. In this study, we proposed a method, called iT3SE-PX, to identify T3SEs solely based on protein sequences. First, three kinds of features were extracted from the position-specific scoring matrix (PSSM) profiles to help train a machine learning (ML) model. Then, the extreme gradient boosting (XGBoost) algorithm was performed to rank these features based on their classification ability. Finally, the optimal features were selected as inputs to a support vector machine (SVM) classifier to predict T3SEs. Based on the two benchmark datasets, we conducted a 100-time randomized 5-fold cross validation (CV) and an independent test, respectively. The experimental results demonstrated that the proposed method achieved superior performance compared to most of the existing methods and could serve as a useful tool for identifying putative T3SEs, given only the sequence information.

## 1. Introduction

The type III secretion systems (T3SSs) are sophisticated protein transport nanomachines that are widely distributed among diverse Gram-negative pathogenic bacteria, including the causative agents of devastating human diseases, such as plague, typhoid fever, and dysentery [1]. Using T3SSs, a variety of virulence proteins are secreted and translocated into host cells, in which they exert a number of effects that help the pathogen to survive and to escape an immune response. These virulence proteins are called type III secreted effectors (T3SEs), which can cause a sequence of changes in host cells, including the subversion of host defences and the modulation of signal transduction pathways [2]. T3SEs vary in number, function, and sequence among different T3SSs or bacterial species, which makes it difficult for identification of T3SEs. Thus, the comprehensive prediction of new T3SEs in pathogenic Gram-negative bacterial proteomes is still a key step towards understanding the molecular mechanisms of host-pathogen interaction and developing better therapeutic targets for critical pathogens. Traditionally, effector proteins are identified and characterized by experimental techniques such as translocation assays [3]. However, conventional experimental methods are often time-consuming and laborious, especially when screening the genome-wide effectors in bacteria. With the development of high-throughput sequencing technology and rapid increase of protein sequence data, there is a growing demand to explore cost-effective computational methods to predict putative T3SEs solely based on their primary sequences.

From the machine learning (ML) perspective, identification of T3SEs is usually described as a binary classification problem. Given a protein sequence as input, ML-based methods automatically predict whether the query protein is a T3SE or not. In recent years, many supervised learning algorithms have been proposed in the literature to solve this problem, including support vector machine (SVM) [4–8], random forest (RF) [9], naive Bayes (NB) [3], artificial neural network (ANN) [10], Markov model [11], latent Dirichlet allocation model [12], ensemble classifiers [13–16], and deep learning [17–19]. The performance of ML-based models depends mainly on the power of their feature encoding schemes. Feature representation numerically formulates diverse-length protein sequences as fixed-length feature vectors, which could be categorized into two groups: (1) N-terminal sequence-based models and (2) full-length sequence-based models.

Previous studies have shown that the first 100 amino acids at the N-terminus of T3SEs may contain important signals that guide their specific recognition by T3SSs [20, 21]. According to this hypothesis, various computational approaches have been applied to predict T3SEs by extracting N-terminal sequence features as inputs of ML-based models [22, 23]. These features usually include amino acid composition (AAC) [22], k-spaced amino acid pair composition [5], certain physic-chemical properties [3], secondary structure [4], solvent accessibility [6], and position-specific scoring matrix (PSSM) profile [9]. For instance, Arnold et al. [3] explored the first ML-based model for predicting T3SEs, called EffectiveT3, by combining AAC and secondary structure of N-terminal sequences. The EffectiveT3 predictor revealed that a strong secretion signal exists in the N-terminus of T3SEs, which can be used to effectively identify T3SEs [3]. Almost simultaneously, Samudrala et al. [8] developed an SIEVE approach to detect T3SEs from genomic protein sequences based on sequence-derived information and to delineate a putative N-terminal secretion signal common to the majority of T3SEs. They also showed that SIEVE can identify known secreted effectors very well with high specificity (SP) and sensitivity (SN) when trained on one species and tested on the other [8]. Then, an SVM-based classifier, called BPBAac, was proposed by Wang et al. for the prediction of T3SEs [22], which extracted the N-terminal position-specific AAC feature by using a Bi-profile Bayes model. The BPBAac classifier outperformed other current implementations in a 5-fold cross validation (CV) and was also robust when tested on a small-size training dataset [22]. A Markov model, namely, T3_MM, was subsequently designed to perform the identification of T3SEs by comparing the total AAC conditional probability difference between N-terminal sequences of T3SEs and non-T3SEs [11]. T3_MM also achieved the more accurate and robust prediction performance when compared with other T3SE recognition algorithms [11]. Dong et al. developed a linear SVM predictor BEAN to identify T3SEs from pathogen genomes by extracting the k-spaced amino acid pair composition from the N-terminal sequences based on the hidden Markov model profiles [23]. Later, Dong et al. presented BEAN 2.0 as an integrated web resource to predict, analyse, and store T3SEs, in which multiple functional analysis tools were provided to assist users in annotating putative T3SEs conveniently [5].

However, recent studies have indicated that some features for accurate effector prediction are contained at the full-length protein sequence level, instead of only residing within the N-terminal region [7, 15]. Goldberg et al. built pEffect as a computational tool to identify T3SEs by combining the sequence similarity-based inference with the SVM-based prediction [7]. The pEffect model not only reached higher performance than existing tools but also suggested for the first time that the recognition signals of T3SEs are distributed over the entire protein sequence and can be picked up by using the local sequence alignment [7]. Recently, a two-layer ensemble predictor Bastion3 was established to accurately classify T3SEs and non-T3SEs from protein sequence data [15]. Bastion3 outperformed several state-of-the-art approaches mainly due to a light gradient boosting machine (LightGBM) used to model training and a wide range of features extracted from three major sources of information, i.e., sequence-based features, physiochemical properties, and evolutionary information [15]. Among these features, the PSSM profile has been shown to provide more important and discriminatory information than sequence itself for various protein function classification tasks such as DNA-binding protein prediction [24], protein structural class identification [25–27], and protein fold recognition [28, 29]. However, the informative features encoded in the PSSM profile have not been adequately explored for the identification of T3SEs in earlier studies.

In this work, we presented a novel predictor, called iT3SE-PX, which further extracted more informative features solely from the PSSM profile to improve the prediction of T3SEs with the help of a powerful feature selection technique. The iT3SE-PX model was designed based on the following four major steps: (i) the PSSM profile of a protein was transformed into a fixed-length feature vector by fusing three feature extraction methods including reduced PSSM (RPSSM), evolutionary difference transformation (EDT), and normalized Moreau-Broto auto correlation (NMBAC); (ii) the hybrid features were scaled into the 0-1 range using the Min-Max normalization; (iii) the extreme gradient boosting (XGBoost) algorithm was adopted as a feature selection technique to rank these features according to their importance; and (iv) a classical SVM learner was used to perform the final prediction of T3SEs based on selected optimal features. The evaluation results indicated that iT3SE-PX performed better on the 100-time 5-fold CV as well as on the independent test compared with existing bioinformatics tools for detecting T3SEs.

## 2. Materials and Methods

**Figure 1:**
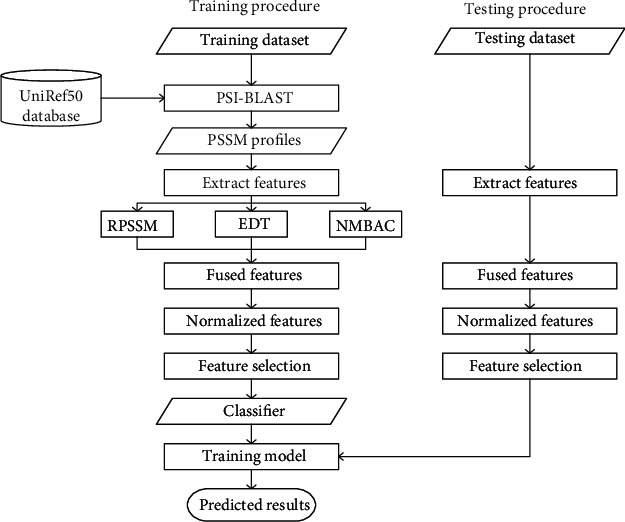
System diagram of the proposed iT3SE-PX model.

In this section, we reported all details of the presented model for the computational recognition of T3SEs based on protein sequence data only. The overall workflow of iT3SE-PX was illustrated in Figure 1. Several important intermediate steps in the design process were explained in detail in the following subsections.

### 2.1. Datasets

To model the task of T3SE identification as an ML problem, the first important step is to establish a comprehensive, reliable, and high-quality benchmark dataset which consists of samples from both positive (T3SEs) and negative (non-T3SEs) classes. In this study, the same dataset constructed by Wang et al. [15] was adopted to evaluate the proposed method. They first collected the training dataset by mining currently known T3SEs from the literature and several existing T3SE databases [5, 30, 31]. Then, they manually removed wrongly annotated effectors and homologous sequences with more than 70% sequence similarity using the CD-HIT program [32]. As a result, the final benchmark dataset contained 379 T3SEs and 1112 non-T3SEs, which were applied for model training and testing by using the 100-time 5-fold CV.

In addition, an independent test dataset which was also built by Wang et al. [15] was used to further rigorously examine the robustness of our predictor and compare it with the existing state-of-the-art T3SE classifiers. The independent dataset consisted of 108 T3SEs and 108 non-T3SEs, which was generated by using the similar strict criteria. They first manually extracted T3SEs from recently published literature and non-T3SEs from various bacterial species and then removed these proteins that have 40% or higher sequence similarity with any protein in the training dataset.

### 2.2. Feature Extraction

#### 2.2.1. PSSM Profiles

Novel T3SEs are usually difficult to identify given that they are very diverse in their AAC and secondary structure elements. Limited prediction accuracies were obtained by sequence-based predictors which only mined characteristics from protein sequences. In contrast, evolutionary features extracted from the PSSM profile can provide more informative patterns and have been widely applied to protein attribute and function classification tasks.

In this work, PSSM profiles were first generated by running the PSI-BLAST program [33] against the UniRef50 database with three iterations and a specified *e*-value score of 0.001. For a query protein with length of *L*, its PSSM profile is an *L* × 20 matrix. The (*i*, *j*)th entry of the resulting matrix denotes the probability score of amino acid type *j* occurring at the *i*th position of the query sequence. Obviously, the higher the score, the more conserved the amino acid at the corresponding position. Each element of the PSSM profile was normalized to the range between 0 and 1 by using the following sigmoid function:

(1) $$\begin{array}{c} f\left(x\right)=\frac{1}{1+e^{-x}}, \end{array}$$

where *x* is the original value of the PSSM profile.

Next, we extracted three types of evolutionary features by exploring information from the PSSM profiles in different aspects, including RPSSM, EDT, and NMBAC.

#### 2.2.2. RPSSM-Based Features

For convenience, we denoted the standardized PSSM of the query sequence as follows:

(2) $$\begin{array}{c} P=\left(P_{A},P_{R},P_{N},P_{D},P_{C},P_{Q},P_{E},P_{G},P_{H},P_{I},P_{L},P_{K},P_{M},P_{F},P_{P},P_{S},P_{T},P_{W},P_{Y},P_{V}\right), \end{array}$$

or

(3) $$\begin{array}{c} P=\left(P_{1},P_{2},\cdots ,P_{20}\right)={\left(p_{i,j}\right)}_{L\times 20}, \end{array}$$

where *P*_*A*_, *P*_*R*_, ⋯, *P*_*V*_ or *P*_1_, *P*_2_, ⋯, *P*_20_ represent the 20 columns in the original PSSM corresponding to the 20 native types of amino acids.

RPSSM is an *L* × 10 matrix by merging some columns of the original PSSM profile [34], which could be denoted as

(4) $$\begin{array}{c} M=\left(M_{1},M_{2},\cdots ,M_{10}\right)={\left(m_{i,j}\right)}_{L\times 10}. \end{array}$$

Here,

(5) $$\begin{array}{c} M_{1}=\frac{P_{F}+P_{Y}+P_{W}}{3},M_{2}=\frac{P_{M}+P_{L}}{2},M_{3}=\frac{P_{I}+P_{V}}{2},M_{4}=\frac{P_{A}+P_{T}+P_{S}}{3},M_{5}=\frac{P_{N}+P_{H}}{2},M_{6}=\frac{P_{Q}+P_{E}+P_{D}}{3},M_{7}=\frac{P_{R}+P_{K}}{2},M_{8}=P_{C},M_{9}=P_{G},M_{10}=P_{P}. \end{array}$$

Then, RPSSM is transformed into a 10-dimensional feature vector by using the following formula:

(6) $$\begin{array}{c} D_{s}=\frac{1}{L}\overset{L}{\underset{i=1}{\sum }}{\left(m_{i,s}-\overset{¯}{m_{s}}\right)}^{2}, \end{array}$$

where

(7) $$\begin{array}{c} \overset{¯}{m_{s}}=\frac{1}{L}\overset{L}{\underset{i=1}{\sum }}m_{i,s}\left(s=1,2,\cdots ,10\right). \end{array}$$

As we all know, sequence-order information is as important as its AAC in a protein sequence. To partially reflect the local sequence-order effect, the pseudo-composition of the gapped dipeptide is introduced to explore the long-range correlation between two residues separated by one or more positions, which can be computed by

(8) $$\begin{array}{c} D_{s,t,\text{lag}}=\frac{1}{L-\text{lag}}\overset{L-\text{lag}}{\underset{i=1}{\sum }}\frac{{\left(m_{i,s}-m_{i+\text{lag},t}\right)}^{2}}{2}\;\left(1\leq s,t\leq 10\right). \end{array}$$

Here, the value of position interval lag ranges from 1 to 10. As a result, we obtained a total of 1010 features extracted from RPSSM by combining *D*_*s*_ and *D*_*s*,*t*,lag_.

#### 2.2.3. Evolutionary Difference Transformation (EDT)

EDT is able to mine the information of the noncooccurrence probability of two residues separated by a certain distance *d* in the any two columns of the PSSM profile [35]. A 400 × *D* − dimensional feature vector could be finally generated as follows:

(9) $$\begin{array}{c} f_{x,y,d}=\frac{1}{L-d}\overset{L-d}{\underset{i=1}{\sum }}{\left(p_{i,x}-p_{i+d,y}\right)}^{2}\;\left(1\leq x,y\leq 20,1\leq d\leq D\right), \end{array}$$

where *p*_*i*,*j*_ represents the value in the *i*th row and *j*th column of the normalized PSSM profile and *D* is the maximum value of *d*. Note that the value of parameter *D* was set to 10 in the subsequent analysis.

#### 2.2.4. Normalized Moreau-Broto Auto Correlation (NMBAC)

NMBAC is a kind of autocorrelation descriptors and has been widely used as a feature encoding technique for the prediction of protein attribute and function, including membrane protein types [36], DNA-binding proteins [37], and protein subnuclear localizations [38]. In this work, we adopted NMBAC to extract the correlation features between two elements within each column of the PSSM profile using the following function:

(10) $$\begin{array}{c} {\text{NMBAC}}_{\text{lag},j}=\frac{1}{L-\text{lag}}\overset{L-\text{lag}}{\underset{i=1}{\sum }}p_{i,j}\times p_{i+\text{lag},j}\;\left(1\leq j\leq 20\right). \end{array}$$

Here, lag denotes the distance between two elements and also ranges from 1 to 10. Finally, a 200-dimensional vector was obtained for each protein sequence.

### 2.3. Feature Normalization

Since the range of values of raw features varies widely, feature scaling is regarded as an essential step towards increasing the ability of the predictive models, especially for the distance-based classifiers. In this study, we adopted the Min-Max normalization method to rescale the raw features into the range between 0 and 1. The Min-Max algorithm maps an original value *X* to the normalized value *X*′, using the following linear transformation:

(11) $$\begin{array}{c} X^{\prime }=\frac{X-X_{\min}}{X_{\max}-X_{\min}}. \end{array}$$

Here, *X*_min_ and *X*_max_ represent the minimum and the maximum values of the variable *X* in the training samples.

### 2.4. Model Construction

#### 2.4.1. Support Vector Machine (SVM)

SVM, which was first proposed by Cortes and Vapnik [39], is one of the most widely used supervised learning algorithms in computational biology, especially suitable for the binary classification tasks [40]. Given a set of labelled training examples, an SVM algorithm learns a linear decision boundary by finding the optimal hyperplane to assign new examples to one category or the other. In addition, SVM can efficiently perform a nonlinear classification when using the kernel trick, implicitly mapping the inputs into high-dimensional feature spaces. In this work, we used the Scikit-learn Python library [41] to construct the SVM-based T3SE predictor based on protein sequence data alone. The radial basis function was chosen as the kernel function due to its excellent performance in the previous applications. We performed a grid search method to optimize the two parameters *C* and *γ* in the search spaces {2^−5^, 2^−3^, 2^−1^, ⋯, 2^15^} and {2^3^, 2^1^, 2^−1^, ⋯, 2^−15^}, and the other parameters were set at the default.

#### 2.4.2. Feature Selection

In ML, feature selection is the process of selecting a subset of most relevant features from the original features for use in model construction. Generally, feature selection techniques can avoid the curse of dimensionality, shorten the training times, and enhance generalization by reducing redundant or irrelevant features without incurring much loss of information. In this work, an XGBoost-based feature selection approach was carried out in an incremental stepwise greedy method [42]. First, we applied the XGBoost classifier to compute an importance score for each feature based on its participation in making key decisions with boosted decision trees. Next, all of features were ranked according to their importance scores. Then, we generated several feature subsets that contained the different top *K* ranked features. Finally, these feature subsets were sequentially input into an ML classifier to select the optimal one. To the best of our knowledge, XGBoost-based feature selection technique has not been used for the identification of T3SEs.

### 2.5. Performance Evaluation

To rigorously and comprehensively evaluate the performance of the proposed model, five widely used standard metrics were reported based on the CV test as well as the independent test, including sensitivity (SN), specificity (SP), accuracy (ACC), *F*-value, and Matthew's correlation coefficient (MCC) [43–46]. These metrics are defined as follows:

(12) $$\begin{array}{c} \text{SN}=\frac{\text{TP}}{\text{TP}+\text{FN}},\\ \text{SP}=\frac{\text{TN}}{\text{TN}+\text{FP}},\\ \text{ACC}=\frac{\text{TP}+\text{TN}}{\text{TP}+\text{FP}+\text{TN}+\text{FN}},\\ F‐\text{value}=\frac{2\text{TP}}{2\text{TP}+\text{FP}+\text{FN}},\\ \text{MCC}=\frac{\text{TP}\times \text{TN}-\text{FP}\times \text{FN}}{\sqrt{\left(\text{TP}+\text{FN}\right)\times \left(\text{TP}+\text{FP}\right)\times \left(\text{TN}+\text{FP}\right)\times \left(\text{TN}+\text{FN}\right)}}, \end{array}$$

where TN, FN, TP, and FP denote the numbers of true negative, false negative, true positive, and false positive, respectively. Besides, the receiver operating characteristic (ROC) curve was adopted to illustrate the diagnostic ability of a binary classifier, which is created by plotting the true positive rate (TPR) against the false positive rate (FPR) at various threshold settings. Note that the TPR is also known as SN, and the FPR can be calculated as 1-SP in ML. The area under the curve (AUC) was also used as a reliable measure. The larger the value of AUC, the better the performance of the predictor.

## 3. Results and Discussion

### 3.1. The Effect of Feature Selection Based on XGBoost

**Figure 2:**
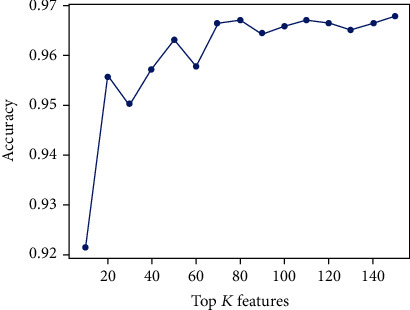
This graph shows how different top *K* features affect the overall accuracies.

In this work, we first obtained a 5210-dimensional feature vector for each protein sequence by performing three feature extraction methods (i.e., RPSSM, EDT, and NMBAC). Although the integrated features captured more sequence information from multiple aspects, the original feature space could contain some redundant or irrelevant features which may lead to the model overfitting and the training time increasing. Therefore, we adopted an XGBoost-based feature selection technique to find the optimal subset of features and improve the prediction performance: (1) less prone to overfitting, (2) much faster, and (3) higher overall accuracy. These features were ranked based on their classification ability, and the top *K* features were examined by the 5-fold CV test, where *K* = 10, 20, 30, ⋯, 150. The results on the training dataset are illustrated in Figure 2. As can be seen, the accuracy achieves a maximum value (96.7%) when *K* increases to 80 and 110. This finding suggested that the most discriminative features from the original feature space could be extracted to form a subset that preserved the original semantics of variables and may be better modelled and interpreted by ML algorithms. In order to select an optimal feature subset for final peptide representation, we constructed two models based on the top 80 features (80D) and the top 110 features (110D) for further analyses.

### 3.2. Performance Comparison of Models Trained Using Different Features

To further evaluate the effectiveness of the proposed XGBoost-based feature selection strategy, we compared the performance of the models trained using different feature encoding methods as well as the selected 80D and 110D features. For each feature type, we trained an individual feature-based SVM model with the optimally tuned parameters and validated its predictive performance by performing a 100-time randomized 5-fold CV test. As shown in Table 1, three individual feature-based models performed well with the ACC of about 95%, which indicated that the informative patterns for identifying T3SEs may be captured by these PSSM-based feature encoding strategies. Particularly, the 80D-based model showed superior overall prediction performance compared with three individual feature-based models and obtained the highest SN value of 92.3%. Moreover, the model trained using the 110D features achieved the best overall performances in term of four metrics: ACC of 96.7%, SP of 98.2%, *F*-value of 93.4%, and MCC of 0.912. This suggested that the selected 80D and 110D-based models not only reduced the training time and the computational complexity but also had reasonable discriminatory power for the prediction of T3SEs.

### 3.3. Performance Comparison with Commonly Used ML Algorithms

In this section, we evaluated the performances of commonly used ML classifiers trained using the selected 110D features, including SVM, *k*-nearest neighbour (KNN), NB, GBM, RF, and XGBoost. To make a fair performance comparison, all experiments were conducted on the same training dataset by using the 100-time 5-fold CV tests. The prediction results are shown in Table 2.

**Figure 3:**
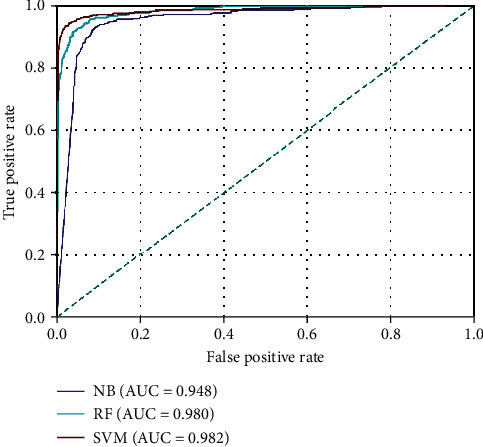
ROC curves of SVM, RF, and NB classifiers based on the 5-fold CV tests. The AUC values were calculated and shown in the inset.

As can be seen, the SVM predictor clearly outperformed the other classifiers in terms of five measures: SN, SP, *F*-value, MCC, and ACC. Moreover, the XGBoost method obtained the second-best predictive performance except that its SN value was just a little lower than those of the KNN and NB classifiers. In comparison, the NB model performed worst in this task. Additionally, the algorithms of KNN, GNM, and RF showed the acceptable performances with the ACC value of larger than 0.94, the MCC value of larger than 0.85, the *F*-value of larger than 0.85, and the SP value of large than 0.96. To assure the distinct and high quality of the target figure, only three ROC curves associated with SVM, RF, and NB models are plotted in Figure 3, which illustrated the consistent conclusions with Table 2. Owing to its accurate prediction power, SVM was adopted as the final predictor for the identification of T3SEs in this work.

### 3.4. Performance Comparison with Existing Methods

In this section, we first compared the performance of the proposed iT3SE-PX model with that of the Bastion3 predictor [15] on the same training dataset by using the 100-time 5-fold CV test. Bastion3 explored a wide range of features from various types such as sequence-based features, physicochemical properties, and evolutionary information [15]. Among these features, five PSSM-based feature encoding methods achieved the top-level performance [15]. To make a fair comparison, the prediction results of our method and only five PSSM-based models in Bastion3 are provided in Table 3.

From Table 3, we observed that the proposed iT3SE-PX model outperformed the listed methods used in Bastion3 in terms of ACC (96.7%), MCC (0.912), and SP (98.2%). Especially, compared with the other five models, iT3SE-PX provided more than 10% improvement in ACC value. It is worth mentioning that the PSSM-composition method achieved the remarkable SN value of 93.0% and the DP-PSSM method achieved the best *F*-value of 94.5%. This means that these algorithms could acquire the important recognition signals from different views and have a mutually supplementary effect. In addition, our method gave the acceptable performance in term of SN (>90%) and *F*-value (>93%) when only 110 features were used. This observation reconfirmed that PSSM-based feature encoding schemes could indeed extract more informative patterns for T3SE identification, and feature selection techniques could help to effectively enhance the performance of T3SE prediction.

To further assess the performance and robustness of the proposed model, we carried out the same independent test with Bastion3 [15], where iT3SE-PX was beforehand trained on the benchmark training dataset.  Table 4 reports five performance measures of iT3SE-PX and seven other state-of-the-art methods on the independent dataset, including Bastion3 [15], BEAN 2.0 [5], pEffect [7], EffectiveT3 [3], T3_MM [11], BPBAac [22], and SIEVE [8].

As shown in Table 4, the iT3SE-PX gained an advantage over other models in terms of ACC (96.3%), MCC (0.927), and *F*-value (96.3%). The resulting SN (94.6%) and SP (98.1%) values ranked next to the best. SIEVE achieved the highest SP values, but SN values were less than 20%, which indicated a tendency to generate more false negatives. In addition, the recently reported Bastion3 model attained the comparable performance with that of our method. We noticed that the Bastion3 applied a two-layer ensemble learning technique to establish a powerful predictor for the identification of T3SEs, which utilized three different types of features. However, our method also obtained satisfactory prediction results when only selected 110D features were used to train an SVM model.

In summary, the proposed method achieved better prediction performance using the relatively few features in comparison with previous studies. However, we should point out that there is still more room for further improvement by exploring multiview features from protein sequences, physicochemical properties, and evolutionary information and developing powerful ensemble classifiers. In the future, we will also develop a user-friendly and publicly accessible online web server of iT3SE-PX to maximize user convenience.

## 4. Conclusions

Despite a dramatic increase in the number of available whole-genome sequences, accurate prediction of T3SEs still remains a challenging problem in bioinformatics. In this work, we proposed an iT3SE-PX model to further improve predictive accuracy of T3SEs solely based on sequence data. First, we integrated three feature extraction techniques (i.e., RPSSM, EDT, and NMBAC) to transform the PSSM profiles of query proteins into 5210-dimensional feature vectors. Then, the XGBoost algorithm was adopted to calculate an importance score for each feature, and all of the features were ranked according to these scores. Finally, the optimal 110 features were selected by using an incremental stepwise greedy method and input into the SVM classifier to perform the prediction of T3SEs. Validation results on two working datasets showed that our method performed better than most of the other existing predictors based on the 100-time 5-fold CV test as well as on the independent dataset test. These promising results also indicated that the proposed iT3SE-PX model could be used for effective prediction of T3SEs, given only the sequence information. For easy implementation, all the datasets and the source codes for this study are freely available to the academic community at https://github.com/taigangliu/iT3SE-PX.

## Figures and Tables

**Table 1:** Performance comparison of different SVM classifiers on the 5-fold CV test.

Feature	SN	SP	*F*-value	MCC	ACC
RPSSM	0.895 ± 0.007	0.976 ± 0.003	0.911 ± 0.005	0.881 ± 0.007	0.955 ± 0.003
EDT	0.910 ± 0.005	0.976 ± 0.002	0.919 ± 0.004	0.891 ± 0.005	0.959 ± 0.002
NMBAC	0.911 ± 0.008	0.965 ± 0.003	0.905 ± 0.006	0.872 ± 0.008	0.951 ± 0.003
80D	0.923 ± 0.006^∗^	0.981 ± 0.002	0.933 ± 0.004	0.911 ± 0.006	0.966 ± 0.002
110D	0.920 ± 0.004	0.982 ± 0.001^∗^	0.934 ± 0.004^∗^	0.912 ± 0.005^∗^	0.967 ± 0.002^∗^

Values were expressed as the mean ± standard deviation. ^∗^The best performance value for each measure (the same below).

**Table 2:** Performance comparison of different classifiers based on the 5-fold CV tests.

Method	SN	SP	*F*-value	MCC	ACC
SVM	0.920 ± 0.004^∗^	0.982 ± 0.001^∗^	0.934 ± 0.004^∗^	0.912 ± 0.005^∗^	0.967 ± 0.002^∗^
KNN	0.913 ± 0.006	0.962 ± 0.003	0.902 ± 0.005	0.869 ± 0.006	0.950 ± 0.002
NB	0.915 ± 0.004	0.913 ± 0.002	0.844 ± 0.003	0.790 ± 0.004	0.914 ± 0.002
GBM	0.900 ± 0.007	0.974 ± 0.002	0.911 ± 0.005	0.881 ± 0.007	0.955 ± 0.002
RF	0.875 ± 0.009	0.967 ± 0.003	0.888 ± 0.007	0.851 ± 0.009	0.943 ± 0.004
XGBoost	0.908 ± 0.006	0.976 ± 0.002	0.917 ± 0.004	0.890 ± 0.006	0.958 ± 0.002

**Table 3:** Performance comparison between iT3SE-PX and Bastion3 on the 5-fold CV test.

Method	SN	SP	*F*-value	MCC	ACC
PSSM-composition	0.930 ± 0.006^∗^	0.949 ± 0.003	0.944 ± 0.003	0.893 ± 0.005	0.857 ± 0.006
RPM-PSSM	0.900 ± 0.008	0.945 ± 0.003	0.933 ± 0.003	0.872 ± 0.007	0.828 ± 0.009
D-FPSSM	0.865 ± 0.010	0.949 ± 0.004	0.927 ± 0.004	0.857 ± 0.008	0.809 ± 0.011
TPC	0.900 ± 0.007	0.953 ± 0.003	0.940 ± 0.003	0.883 ± 0.006	0.843 ± 0.008
DP-PSSM	0.925 ± 0.007	0.952 ± 0.003	0.945 ± 0.003^∗^	0.894 ± 0.005	0.858 ± 0.007
iT3SE-PX	0.920 ± 0.004	0.982 ± 0.001^∗^	0.934 ± 0.004	0.912 ± 0.005^∗^	0.967 ± 0.002^∗^

**Table 4:** Performance comparison on the independent test.

Method	SN	SP	*F*-value	MCC	ACC
iT3SE-PX	0.946 ± 0.043	0.981 ± 0.036	0.963 ± 0.035^∗^	0.927 ± 0.069^∗^	0.963 ± 0.034^∗^
Bastion3	0.954 ± 0.034^∗^	0.958 ± 0.025	0.958 ± 0.010	0.917 ± 0.017	0.959 ± 0.008
BEAN 2.0	0.891 ± 0.060	0.917 ± 0.069	0.906 ± 0.024	0.816 ± 0.033	0.908 ± 0.015
pEffect	0.878 ± 0.088	0.909 ± 0.041	0.889 ± 0.066	0.790 ± 0.100	0.895 ± 0.048
EffectiveT3	0.741 ± 0.086	0.873 ± 0.037	0.794 ± 0.051	0.623 ± 0.068	0.809 ± 0.038
T3_MM	0.804 ± 0.04	0.783 ± 0.054	0.797 ± 0.043	0.588 ± 0.066	0.795 ± 0.031
BPBAac	0.288 ± 0.067	0.978 ± 0.031	0.437 ± 0.082	0.371 ± 0.072	0.629 ± 0.062
SIEVE	0.122 ± 0.057	1.000 ± 0.000^∗^	0.214 ± 0.091	0.247 ± 0.063	0.557 ± 0.048

## Data Availability

The data used to support the findings of this study are freely available to the academic community at https://github.com/taigangliu/iT3SE-PX.
